# Supplementary material for: Autism spectrum disorder in young patients with congenital central hypoventilation syndrome: role of the autonomic nervous system dysfunction
Source: Orphanet J Rare Dis. 2024 Jul 3;19:249. doi: 10.1186/s13023-024-03257-z (PMC11220943; doi:10.1186/s13023-024-03257-z)
Supplement: Supplementary file 1 — Supplementary Material 1 [file 13023_2024_3257_MOESM1_ESM.docx]

**Autism spectrum disorder in young patients with congenital central hypoventilation syndrome: role of the autonomic nervous system dysfunction**

Benjamin Dudoignon^1^ MD, Anna Maruani^2^ MD, PhD, Richard Delorme^2^ MD, PhD, Maxime Patout^3^ MD, PhD, Mylene Fefeu^2^ MD, PhD, Pierre Ellul^2^ MD, PhD Plamen Bokov^1^ MD, PhD, Christophe Delclaux^1^ MD, PhD

^1^: Université de Paris-Cité, AP-HP, Hôpital Robert Debré, Service de Physiologie Pédiatrique-Centre du Sommeil – CRMR Maladies respiratoires rares – Hypoventilations alvéolaires rares – Syndrome d’Ondine, INSERM NeuroDiderot, F-75019 Paris, France

^2^: Université de Paris-Cité, AP-HP, Hôpital Robert Debré, Service de psychiatrie de l’enfant et l’adolescent 75 019 Paris France

^3^: Sorbonne Université, AP-HP, Hôpital de la Pitié-Salpêtrière, Service des Pathologies du Sommeil (Département R3S) - CRMR Hypoventilations centrales congénitales, INSERM, UMRS1158 Neurophysiologie Respiratoire Expérimentale et Clinique, F-75005 Paris, France

Corresponding author: Benjamin Dudoignon

Service de Physiologie – Explorations Fonctionnelles

Hôpital universitaire pédiatrique Robert Debré

48, boulevard Sérurier

75019 Paris

Email: [benjamin.dudoignon@aphp.fr](mailto:benjamin.dudoignon@aphp.fr)

**Abstract**

**Background.** Congenital central hypoventilation syndrome (CCHS) is a rare condition characterized by alveolar hypoventilation and autonomic nervous system (ANS) dysfunction requiring long-term ventilation. CCHS could constitute a risk factor of autism spectrum disorder (ASD) due to birth injury related to respiratory failure, which remains to be determined. ANS dysfunction has also been described in ASD and there are indications for altered contribution of ANS-central nervous system interaction in processing of social information; thus, CCHS could be a risk factor for ASD based on pathophysiological background also. Our study aimed to determine the prevalence of ASD among CCHS patients, identify risk factors, and explore the relationship between the ANS, evaluated by heart rate variability indices, and adaptative functioning.

**Results**. Our retrospective study, based on the analysis of records of a French national center of patients with CCHS under 20 years of age, determined that the prevalence of ASD (diagnosed by a psychiatrist, following the criteria of DSM-4 or DSM-5) was 6/69 patients, 8.7% (95% confidence interval: 3.3 – 18.0 %). In a case (CCHS with ASD, n=6) – control (CCHS without ASD, n=12) study with matching on sex, longer neonatal hospitalization stay and glycemic dysfunction were associated with ASD. Adaptative functioning was assessed using Vineland Adaptative behavioral scales (VABS) and heart rate variability indices (including daytime RMSSD as an index of parasympathetic modulation) were obtained from ECG Holter performed the same day. In 19 young subjects with CCHS who had both ECG Holter and VABS, significant positive correlations were observed between RMSSD and three of four sub-domains of the VABS (communication: R=0.50, p=0.028; daily living skills: R=0.60, p=0.006; socialization: R=0.52, p=0.021).

**Conclusion**. Our study suggests a high prevalence of ASD in patients with CCHS. Glycemic dysfunction and longer initial hospitalization stays were associated with ASD development. A defect in parasympathetic modulation was associated with worse adaptative functioning.

**Keywords**

Congenital central hypoventilation syndrome, Autism spectrum disorder, Heart rate variability, Autonomic nervous system

**Introduction**

Congenital central hypoventilation syndrome (CCHS) is a rare disorder characterized by alveolar hypoventilation requiring long-term invasive or noninvasive ventilation and autonomic nervous system (ANS) dysfunction [1]. *PHOX2B* was identified as the major CCHS-causing gene, and different mutations have been reported: in-frame tandem duplications of tracts of different lengths of the polyalanine stretch in exon 3 (polyalanine repeat mutations, PARM) are the most frequent [1], and the severity of ANS dysfunction has been related to the number of gene expansions [1]. *PHOX2B* is essential for the development of autonomic neural crest derivatives [2], explaining the generalized ANS dysfunction in CCHS. As recommended by guidelines [1], a comprehensive assessment of this dysfunction is undertaken at diagnosis, involving mainly the cardiovascular, digestive, and ocular systems, as well as the metabolic and endocrine status. The neurodevelopmental and neurocognitive status are also assessed. The hospital discharge is usually long, and the initial management of these children takes place in neonatal or pediatric intensive care with a multidisciplinary approach. Neurodevelopmental disorders (developmental delays, formal diagnosis of learning disabilities, attention deficit hyperactivity disorder) were observed in a survey of 196 patients with CCHS [3]. Several studies have described neurocognition in this particular population [4–6]. One case report described a severe developmental delay with autism spectrum disorder (ASD) [7], and three patients were reported with ASD or pervasive developmental disorder [3]. ASD is a set of neurodevelopmental conditions characterized by early-onset difficulties in social communication and interaction and restricted, repetitive behavior and interest according to the Diagnostic and Statistical Manual of Mental Disorders (DSM)-5 criteria [8]. The worldwide prevalence is about 1% [8]. Factors associated with ASD risk are abnormal presentation, umbilical cord complications, fetal distress, birth injury or trauma, multiple births, maternal hemorrhage, summer birth, low birth weight, small for gestational age, congenital malformation, low 5-minute Apgar score, and feeding difficulties among other factors [9]. Thus, CCHS could also constitute a risk factor for ASD due to a birth injury related to respiratory failure [10].

ANS dysfunction has also been described in ASD, and there are indications for an altered contribution of ANS–central nervous system interaction in processing social information [11]. A defect in parasympathetic modulation can even be present before ASD diagnosis [12], which may contribute to an increase in the prevalence of ASD in children with CCHS due to their generalized defect in parasympathetic modulation.

The aim of our study was to define the prevalence of ASD within the French national CCHS reference center, to describe the post-natal risk factors, and to further assess the relationship in children with CCHS between ANS dysfunction evaluated by heart rate variability (HRV) analysis and adaptative functioning assessed by the Vineland Adaptive Behavior Scales (VABS). We hypothesized that the ASD prevalence would be increased in CCHS and associated with generalized ANS dysfunction, including heart rate and the central nervous system.

**Material and Methods**

**Design**

This retrospective study was based on the analysis of medical records. Ethical approval was obtained from our local Ethical Committee to collect data from this cohort (CEER-629-2022).

*First part of the study: prevalence of ASD in patients with CCHS*

From the medical records of all children and young patients with CCHS followed up in our national reference center (n = 69), the prevalence of ASD was estimated (patients with ASD) versus those without on 07/25/2023. The inclusion criterion comprised children under the age of 20 who were diagnosed with CCHS according to international definition [1]. ASD was diagnosed by a psychiatrist, following the diagnostic criteria outlined in either DSM-4 or DSM-5. Scales such as Vineland Adaptative Behavior Scales II (VABS-II), Autism Diagnosis Observation Schedule 2^nd^ edition (ADOS-2), Childhood Autism Rating Scale (CARS), or Autism Diagnostic Interview (ADI) were recorded. Regarding cognitive function, patients underwent cognitive tests during their Robert Debré hospitalization when the test was feasible (Wechsler test).

Subjects were classified as having moderate PARM (PARM-25, PARM-26) or severe mutations (PARM≥27 or non-PARM variants).

*Second part of the study*

Children with ASD and CCHS were matched (1:2 ratio) with children with CCHS without ASD for sex since male predominance of ASD is established and since sex differences in clinical presentations point to the possibility that the methods used for the diagnosis of children with ASD may be more appropriate for boys [13].

*Third part of the study*

We hypothesized that a relationship could be evidenced between heart ANS dysfunction and autonomic central nervous system dysfunction (adaptative functioning), explaining the high prevalence of ASD in children with CCHS.

The VABS-II assessed adaptative functioning, with scores ranging from 20 to 160; higher scores indicated better functioning [14]. The VABS has an age-corrected mean score of 100 and a standard deviation of 15. VABS has four sub-domains: communication, daily living skills, socialization, and motor skills. The communication domain comprises receptive, expressive, and written subscales; the daily living skills domain comprises personal, domestic, and community subscales; the socialization domain comprises interpersonal relationships, play and leisure, and coping skills subscales; and the motor skills domain comprises gross and fine motor subscales. The VABS-III has been linked to both the Comprehensive International Classification of Functioning, Disability and Health Core Set for Autism and the Brief International Classification of Functioning, Disability and Health Core Set for Autism [15].

**Ambulatory ECG monitoring (ECG Holter)**

According to CCHS guidelines [1], a 48-h ambulatory ECG monitoring was done, from which we selected the 24-h ambulatory ECG monitoring obtained during the same day of neurobehavioral function assessment. The electrocardiographic recording was acquired with the Holter recorder SpiderView (ELA Medical, SORIN Group, Clamart, France). Analog data were edited on a SyneScope station and exported in ASCII files. The files were then processed using the HRV analysis software 1.1, downloaded at [https://anslabtools.univ-st-etienne.fr](https://anslabtools.univ-st-etienne.fr/), and validated by Pichot et al. [16]~~, as previously done [17]~~. The 24-h recordings were split into daytime (8 AM–9 PM) and nighttime (11 PM–6 AM) as previously done [17]. Time domain variables included the mean sinus heart rate (HR), the standard deviation of the RR intervals (SDNN), and the root mean of squared successive differences (RMSSD: an index of parasympathetic modulation). After fast Fourier transform, the power spectrum indices were calculated, as previously described [17]. The frequency-domain measurements were the very low frequencies (VLF: 0–0.04 Hz), low frequencies (LF: 0.04–0.15 Hz), and high frequencies (HF: 0.15–0.40 Hz). LF and HF were also expressed as normalized values, LFnu and HFnu, and the LF/HF ratio was calculated. HF is an index of parasympathetic modulation, while higher values of LF/HF ratio are related to increased sympathetic modulation, due to the increase of the latter and/or the decrease in parasympathetic modulation. The daytime indices were recorded.

**Statistical analysis**

Results were expressed as median [25^th^–75^th^ percentiles]. Comparisons of continuous variables between groups were performed using the Mann–Whitney U test. Categorical variables were compared using the chi-square test (Fisher’s exact test when necessary). We evaluated correlations by using Pearson’s correlation coefficient. The significance level was 0.05. We used StatView 5.0 (SAS Institute, Cary, NC, USA) for statistical analyses.

**Results**

*First part: prevalence of ASD*

The prevalence of ASD in our cohort was 6/69, 8.7% (95% confidence interval: 3.3–18.0%).

*Second part: case-control study of patients with CCHS with and without ASD*

We included 6 patients with CCHS and ASD and 12 patients with CCHS without ASD, matched for sex. Their characteristics are described in Table 1. Ventilator adherence was similar in both groups. Five of six CCHS patients with ASD had a severe mutation [≥ 20/27 and non-polyalanine repeat mutation (NPARM)]. The genotype moderate (20/25 and 20/26) versus severe does not appear to contribute to the presence of ASD (1/5 versus 8/4, *p* = 0.131, Fisher’s exact test). The duration of the initial hospitalization was higher in children with ASD. One late-onset patient exhibited ASD symptoms prior to receiving a CCHS diagnosis. A detailed description of ASD symptoms and tests of the six children with ASD is provided in Table 2.

*Third part: relationships between an index of parasympathetic modulation and adaptative functioning*

We selected 19 young patients with CCHS from our database who had both an ECG Holter and a neuropsychological assessment during hospitalization that are described in Table 3. Among these 19 patients with CCHS, 10 children with CCHS (4 with ASD diagnosis and 6 without ASD diagnosis) were already included in the first part of the study, and 9 additional patients with CCHS and without ASD were included. The median age of the 19 children was 6.4 years [5.0; 13.2], and their sex ratio was 10 females and 9 males.

This table shows that children with CCHS and ASD, as compared to those without ASD, were characterized by an increased sympathetic modulation due to both sympathetic gain and parasympathetic defect, and by decreased VABS domains (with the exception of motor skills).

The relationships between daytime RMSSD and the four sub-domains of the VABS scale are described in Figure 1. Similar relationships were observed using the LF/HF ratio: communication (*r* = -0.52, *p* = 0.021), daily living skills (*r* = -0.47, *p* = 0.044), socialization (*r* = -0.56, *p* = 0.012), but not with motor skills (*r* = 0.08, *p* = 0.813). The RMSSD did not significantly correlate with age (*r* = -0.34, *p* = 0.152). The best correlation was observed with the domain of daily living skills; the personal, domestic, and community sub-domains correlated with the RMSSD: *r* = 0.59, *p* = 0.008; *r* = 0.61, *p* = 0.005; *r* = 0.52, *p* = 0.023, respectively. The SDNN did not significantly correlate with any of the four sub-domains of VABS (data not shown). Age negatively significantly correlated with the first three domains of the VABS: communication (*r* = -0.54, *p* = 0.017), daily living skills (*r* = -0.54, *p* = 0.016), socialization (*r* = -0.66, *p* = 0.002), but not with motor skills (*r* = 0.15, *p* = 0.649, n = 11). In the 15 young patients with CCHS and PARM, no significant relationships were observed between the number of PARM and any of the four sub-domains of VABS (data not shown).

**Discussion**

~~The main result of this retrospective study is to suggest that ANS dysfunction of CCHS, namely parasympathetic modulation defect, could partly be responsible for the possible increase in ASD prevalence in CCHS.~~

The prevalence of ASD in the cohort of the French national CCHS center seems higher than in the general population in Europe ranging between 0.38 and 1.55% [18]. The initial symptoms of ASD and the diagnostic delay were similar between our patients and those reported in the literature [19,20]. The duration of the initial hospitalization and the presence of glycemic dysfunction distinguished CCHS children with and without ASD. The duration of initial hospitalization was probably related to the severity of the CCHS, and fetal distress or feeding difficulties have already been identified as risk factors for ASD [9]. Accordingly, many autonomic characteristics of CCHS reflect parasympathetic alterations, including disturbed alimentary processes, with malabsorption and intestinal motility dyscontrol [1]. The relationship with glycemic dysfunction (hypo or hyperglycemia) is also symptomatic of the severity of ANS dysfunction.

The main result of this retrospective study is to suggest that ANS dysfunction of CCHS, namely parasympathetic modulation defect, could partly be responsible for the possible increase in ASD prevalence in CCHS. CCHS and ASD are both characterized by a defect in parasympathetic modulation [17,21]. ~~There were indications for the altered contribution of ANS–central nervous system interaction in processing social information in ASD~~ ANS pathways target several cortical areas (e.g., medial prefrontal cortex, anterior cingulate cortex, anterior insular cortex), some of which also seem to be relevant for processing of socially relevant information [11]. Thus, an increased prevalence of ASD in CCHS may seem expected if central nervous ~~ANS~~ dysfunction is responsible for some ASD symptoms. Along this line, a study that measured ANS response to a peer-based social interaction paradigm in 50 typically developing children and 50 children with ASD showed that parasympathetic functioning may be especially important in behavioral regulation, as older youth with ASD demonstrated atypical regulation and response to the social interaction paradigm [22]. The defect in parasympathetic modulation can even be present before ASD diagnosis, as suggested by a study showing that lower respiratory sinus arrhythmia (a parasympathetic index of HRV analysis) in 4-month-old infants later diagnosed with ASD exhibited poorer autonomic regulation during interaction with an unfamiliar adult than did controls [12]. Finally, there is some evidence that vagal nerve stimulation, when performed for epilepsy, may improve behavior in people with ASD [10]. This background justified the third part of our retrospective study devoted to assessing the relationships between parasympathetic modulation (RMSSD, an index of HRV) and adaptative functioning. The significant relationships evidenced in Figure 1 between RMSSD and three domains of the VABS show that the higher the deficit in parasympathetic modulation is, the worse the adaptative functioning is.

One of the most common disparities in ASD development involves the amygdala, which regulates emotions, particularly fear and anxiety [22]. Individuals with CCHS may have reduced anxiety [23], and 54% of the patients with CCHS showed difficulties in social interactions [23]. Furthermore, parents reported adaptive function problems affecting communication and daily living skills. Thus, the prevalence of ASD could be even higher than we presently report. Logically, recent guidelines state that psychomotor development should be assessed in the first year of life and that comprehensive neurocognitive follow-up should be repeated at least annually in the first few years to plan for special educational needs and/or psychological support [1].

The use of tracheostomy ventilation, which is the most effective ventilation mode for improving nocturnal gas exchange, argues against the role of hypoxia contributing to ASD in our series. Patients with CCHS show reduced gray matter volume with age progression in autonomic, respiratory, and cognitive regulatory areas. This outcome may contribute to the deterioration of functions with increasing age, possibly attributed to repeated hypoxic events [24]. The aggravation of three sub-domains of VABS with age may suggest that hypoxia or hypercapnia may contribute to neurobehavioral function deterioration. However, hypoxia is not supposed to occur once nighttime ventilation (as in all the presently reported children) is started. Along this line, a relationship between neurocognition and severity of respiratory deficit has not been demonstrated in CCHS in the United States [25]. Knowing the role of central autonomic networks on brain functioning, one may wonder to what extent CCHS-related ANS dysfunction may impact the modulation of brain development and plasticity, especially during critical periods of childhood. Our results argue for a role of the loss of parasympathetic modulation. Negative correlations have previously been evidenced between intellectual measures and the number of polyalanine expansions, with significance obtained for indices of working memory and fluid reasoning [4]. In our study, the number of polyalanine expansions did not correlate with the four domains of VABS.

The primary limitations of the study were the small number of patients included, which may explain the absence of a link between the severity of mutations and ASD prevalence and its retrospective nature. Nevertheless, the degree of significance of the relationships between parasympathetic modulation and the three domains of VABS, obtained in a restricted number of patients (n = 19), strongly supports the role of CCHS-related ANS dysfunction in CCHS symptoms.

**Conclusion**

Our study suggests a high prevalence of ASD in patients with CCHS. Glycemic dysfunction and longer initial hospitalization stay were associated with ASD development in this particular population, which probably reflect the severity of ANS dysfunction. The defect in parasympathetic modulation of HR correlated with defective neurobehavioral function in CCHS.

**Abbreviations**

ADI: Autism Diagnostic Interview

ADOS-2: Autism Diagnosis Observation Schedule 2^nd^ edition

ANS: Autonomic nervous system

ASD: Autism Spectrum Disorder

CARS: Childhood Autism Rating scale

CCHS: Congenital central hypoventilation syndrome

DSM: Diagnostic and Statistical Manual of Mental Disorders

HRV: Heart rate variability

NPARM: Non-polyalanine repeat mutation

RMSSD: Root mean of squared successive differences

VABS: Vineland Adaptative Behavioral Scales

**Declarations**

**Data availability statement ~~of data and materials~~**

The data that support the findings of this study are available upon reasonable request to protect study participant privacy

**Ethics approval and consent to participate**

This study was performed in line with the principles of the declaration of Helsinki. Approval was granted by local Ethical Committee to collect data (CEER-629-2022).

**Consent for publication**

All authors consent for publication

**Competing interests**

The authors declare that they have no competing interests

**Funding**

The authors declare that no funds, grants, or other support were received during the preparation of this manuscript

**Authors contributions**

BD, PB, CD designed the study. Material preparation, data collection and analysis were performed by BD and CD. The first draft of the manuscript was written by BD and CD and all authors AM, RD, MP, MF, PE, PB commented on previous versions of the manuscript. All authors read and approved the final manuscript.

**Acknowledgments**

The authors thank the children and parents who have consented to their data being used for research purposes; they also thank the Association Française du Syndrome d'Ondine (AFSO).

**References**

1. Trang H, Samuels M, Ceccherini I, Frerick M, Garcia-Teresa MA, Peters J, et al. Guidelines for diagnosis and management of congenital central hypoventilation syndrome. Orphanet J Rare Dis. 2020;15:252.

2. Pattyn A, Morin X, Cremer H, Goridis C, Brunet JF. The homeobox gene Phox2b is essential for the development of autonomic neural crest derivatives. Nature. 1999;399:366–70.

3. Vanderlaan M, Holbrook CR, Wang M, Tuell A, Gozal D. Epidemiologic survey of 196 patients with congenital central hypoventilation syndrome. Pediatr Pulmonol. 2004;37:217–29.

4. Trang H, Bourgeois P, Cheliout-Heraut F. Neurocognition in Congenital Central Hypoventilation Syndrome: influence of genotype and ventilation method. Orphanet J Rare Dis. 2020;15:322.

5. Charnay AJ, Antisdel-Lomaglio JE, Zelko FA, Rand CM, Le M, Gordon SC, et al. Congenital Central Hypoventilation Syndrome: Neurocognition Already Reduced in Preschool-Aged Children. Chest. 2016;149:809–15.

6. Macdonald KT, Mosquera RA, Yadav A, Caldas-Vasquez MC, Emanuel H, Rennie K. Neurocognitive functioning in individuals with congenital central hypoventilation syndrome. BMC Pediatr. 2020;20:194.

7. Seijas-Gomez R, Esteso-Orduna B, Melero-Llorente J, Fournier-Del Castillo MC. [Clinical and neuropsychological characteristics in congenital central hypoventilation syndrome]. Rev Neurol. 2018;66:303–7.

8. Lai M-C, Lombardo MV, Baron-Cohen S. Autism. Lancet Lond Engl. 2014;383:896–910.

9. Gardener H, Spiegelman D, Buka SL. Perinatal and Neonatal Risk Factors for Autism: A Comprehensive Meta-analysis. Pediatrics. 2011;128:344.

10. van Hoorn A, Carpenter T, Oak K, Laugharne R, Ring H, Shankar R. Neuromodulation of autism spectrum disorders using vagal nerve stimulation. J Clin Neurosci Off J Neurosurg Soc Australas. 2019;63:8–12.

11. Karjalainen S, Aro T, Parviainen T. Coactivation of Autonomic and Central Nervous Systems During Processing of Socially Relevant Information in Autism Spectrum Disorder: A Systematic Review. Neuropsychol Rev. 2023;

12. McCormick CEB, Sheinkopf SJ, Levine TP, LaGasse LL, Tronick E, Lester BL. Diminished respiratory sinus arrhythmia response in infants later diagnosed with autism spectrum disorder. Autism Res Off J Int Soc Autism Res. 2018;11:726–31.

13. Ornoy A, Gorobets D, Weinstein-Fudim L, Becker M. Sex-Related Changes in the Clinical, Genetic, Electrophysiological, Connectivity, and Molecular Presentations of ASD: A Comparison between Human and Animal Models of ASD with Reference to Our Data. Int J Mol Sci. 2023;24:3287.

14. Sparrow SS, Cicchetti DV. The Vineland Adaptive Behavior Scales. Major Psychol Assess Instrum Vol 2. Needham Heights, MA, US: Allyn & Bacon; 1989. p. 199–231.

15. Hayden-Evans M, Milbourn B, D’Arcy E, Chamberlain A, Afsharnejad B, Evans K, et al. An Evaluation of the Overall Utility of Measures of Functioning Suitable for School-Aged Children on the Autism Spectrum: A Scoping Review. Int J Environ Res Public Health. 2022;19:14114.

16. Pichot V, Roche F, Celle S, Barthélémy J-C, Chouchou F. HRVanalysis: A Free Software for Analyzing Cardiac Autonomic Activity. Front Physiol. 2016;7:557.

17. Dudoignon B, Denjoy I, Patout M, Matrot B, Gallego J, Bokov P, et al. Heart rate variability in congenital central hypoventilation syndrome: relationships with hypertension and sinus pauses. Pediatr Res. 2023;93:1003–9.

18. Bougeard C, Picarel-Blanchot F, Schmid R, Campbell R, Buitelaar J. Prevalence of Autism Spectrum Disorder and Co-Morbidities in Children and Adolescents: A Systematic Literature Review. Focus Am Psychiatr Publ. 2024;22:212–28.

19. Daniels AM, Mandell DS. Explaining differences in age at autism spectrum disorder diagnosis: a critical review. Autism Int J Res Pract. 2014;18:583–97.

20. Guthrie W, Swineford LB, Nottke C, Wetherby AM. Early diagnosis of autism spectrum disorder: stability and change in clinical diagnosis and symptom presentation. J Child Psychol Psychiatry. 2013;54:582–90.

21. Beopoulos A, Géa M, Fasano A, Iris F. Autism spectrum disorders pathogenesis: Toward a comprehensive model based on neuroanatomic and neurodevelopment considerations. Front Neurosci. 2022;16:988735.

22. Beopoulos A, Géa M, Fasano A, Iris F. Autism spectrum disorders pathogenesis: Toward a comprehensive model based on neuroanatomic and neurodevelopment considerations. Front Neurosci. 2022;16:988735.

23. Montirosso R, Morandi F, D’Aloisio C, Berna A, Provenzi L, Borgatti R. International Classification of Functioning, Disability and Health in children with congenital central hypoventilation syndrome. Disabil Rehabil. 2009;31 Suppl 1:S144-152.

24. Kumar R, Woo MS, Macey PM, Woo MA, Harper RM. Progressive gray matter changes in patients with congenital central hypoventilation syndrome. Pediatr Res. 2012;71:701–6.

25. Zelko FA, Nelson MN, Leurgans SE, Berry-Kravis EM, Weese-Mayer DE. Congenital central hypoventilation syndrome: neurocognitive functioning in school age children. Pediatr Pulmonol. 2010;45:92–8.
